# Parenting Style and Reactive and Proactive Adolescent Violence: Evidence from Spain

**DOI:** 10.3390/ijerph15122634

**Published:** 2018-11-24

**Authors:** David Moreno-Ruiz, Estefanía Estévez, Teresa I. Jiménez, Sergio Murgui

**Affiliations:** 1 Departamento de Psicología Social, Universidad de Valencia, Avda. Blasco Ibáñez, 13., 46010 Valencia, Spain; sergio.murgui@uv.es; 2 Departamento de Psicología de la Salud, Universidad Miguel Hernández de Elche, Avda. De la Universidad s/n, Edificio Altamira, 03202 Elche (Alicante), Spain; eestevez@umh.es; 3 Departamento de Psicología y Sociología, Universidad de Zaragoza, Ciudad Escolar s/n, 44003 Teruel, Spain; tijimgut@unizar.es

**Keywords:** parenting styles, proactive/reactive violence, adolescence

## Abstract

The aim of the present study was to analyze the relationship between the parenting styles—authoritative, indulgent, authoritarian, and neglectful—and reactive and proactive school violence among peers. These analyses were also performed by sex and age groups. The sample consisted of 2399 Spanish adolescents (50.2% male), aged between 12 and 18 years, mean (M) = 14.69, standard deviation (*SD*) = 1.82. A multivariate analisys of variance (MANOVA, 4 × 2 × 2) was applied, considering parenting style, sex, and age group (12–14 and 15–18 years) as independent variables to analyze the possible effects of interaction. Reactive, proactive, and pure violence were the dependent variables. The results showed the main effects of parenting styles, sex, and age, as well as an interaction between sex, age, and parenting styles. The interpretation of the findings suggested that the authoritarian parenting style was related to greater engagement in proactive and reactive violent behaviors. In relation to the effect of the interaction between sex, age, and parenting styles, it was observed that adolescents from indulgent families, of both sexes and in any of the studied age groups, obtained lower scores in proactive violence. The discussion highlights the importance of affection and warmth for well-adjusted children’s behavior.

## 1. Introduction

This paper examines the relationship between parenting style and school violence in Spanish adolescents. More specifically, we researched how the four parenting styles—authoritarian, neglectful, authoritative, and indulgent—are differentially associated with different functions of violence (reactive or proactive), also taking into account the sex and age of the adolescent participants in this research.

Previous research has examined the relationship between parenting styles and school violence in adolescents (for reviews, see) [1,2,3]. However, although these prior works are suggestive and interesting, research on parenting styles and functions of violence is still incipient, especially regarding the typology proposed by Maccoby et al. [4], which particularly take into account the population of Spain. In addition to this aspect, which in itself constitutes a novelty, it is relevant to explore the role of sex and age, variables narrowly studied in relation to parenting styles and the functions of violence, in order to develop more specific preventive and intervention strategies in each case.

### 1.1. Concept and Functions of Violence

Interest in the study of school violence began in the north of Europe, in the 1970s, with investigations and prevention proposals developed by the Scandinavian researchers, Olweus [5] and Heinemann [6]. Since then, a large amount of studies on school violence has been generated as a response to the growing social concern for the problems of coexistence in the classroom (for reviews, see) [7,8]. This fact, coupled with the negative consequences of violence for the psychosocial development of children and adolescents, justifies the importance of further research on this object of study.

In addition, it is important to conceptualize violent behavior in detail. In this regard, Little, Henrich et al. [9] classified violent acts as two main categories, comprising both the form and the function of this behavior. According to its form, violence can be classified as overt versus relational. Overt violence refers to behaviors that involve a direct confrontation with others with the intention of causing harm (pushing, hitting, threatening, insulting, etc.). Relational violence does not imply a direct confrontation between the aggressor and the victim, and is defined as the act that aims to cause harm in the circle of friends of another person or in their perception of group membership (social exclusion, social rejection, spreading rumors, etc.).

In relation to the function of violent behavior, a central aspect of this work, two fundamental types are observed: Reactive versus proactive violence. Reactive violence refers to behaviors involving a defensive response to some provocation. This violent behavior can be explained through mechanisms of frustration–aggression, and is usually linked to issues of impulsivity and self-control, and a bias in the interpretation of social relations that is based on the tendency to make hostile attributions to others’ behavior [10]. Proactive or instrumental violence refers to goal-driven behavior, that is, the violent act is the instrument through which the aggressor expects to achieve something. This type of violence has been linked to the social learning theory. In this regard, proactive violence can be acquired by observation of models or by direct experience, and involves the anticipation of benefits; it is deliberate and controlled by external reinforcements [11]. This type of behavior has been related to subsequent problems of delinquency, but also to high levels of social competence and leadership skills [12]. The present study also evaluated the pure dimension of violence, referring to an individual’s violent behavior, both overt and relational, regardless of its function [9].

The operationalization of violence established by Little et al. [9] has proven to be very useful in the study of violent school behavior, due to the detailed level of analysis of this variable and how it relates to other relevant psychosocial factors in this problem [13,14,15]. In fact, the development of school violence is considered a complex and multifactorial process [16]. Studies adopting an ecological perspective stress the importance of analyzing different individual (i.e., sex and age) and contextual (i.e., parenting style) factors that contribute to a more precise explanation of violent dynamics in the classroom [17,18].

### 1.2. School Violence and Parenting Styles

Among the contextual factors analyzed in relation to violent behavior at school, different investigations have highlighted those associated with the family environment. During adolescence, peer social relations and the worries arising from them acquire greater relevance than in earlier life stages. However, the importance of the family is not diluted but, instead, remains an essential reference and determining factor at this developmental stage and in the children’s psychosocial well-being [19,20,21]. The family interaction system sets the stage that determines the repertoire of adolescents’ behaviors, and their greater or smaller possibilities of being violent.

In many works, adolescents’ maladaptive and violent behavior is associated with inappropriate educational practices, ineffective supervision and parental guidance, and with negative relationships between family members [8,22,23,24]. Moreover, bullying and cyberbullying have been linked with higher levels of parental stress, and with authoritarian and more permissive practices [25,26,27]. In this sense, the families of students involved in violent behavior at school have great difficulty in controlling their children’s behavior. On the contrary, adolescents who feel emotionally involved with their family show increased confidence and independence and a lower tendency to commit violent and antisocial acts [28].

A family variable of great importance in the child–parent relationship is parental socialization. The dimensions of control–imposition and involvement–acceptance have been identified as being characteristic of parenting styles. The dimension involvement–acceptance is defined as the degree to which parents are emotionally involved in the education of their children, offering them affection and support, and using dialogue and positive communication. The control–imposition dimension refers to the degree to which parents act strictly and negatively to delimit and regulate their children’s behavior through their authority [29].

Based on these two orthogonal dimensions are four key parenting styles: Authoritarian, characterized by high levels of control and low levels of affection; indulgent, which is identified by low control and high affection; authoritative, which employs high levels of control and affection; and, neglectful which is characterized by low levels of control and affection [30,31].

Research of the relations between the different parenting styles and psychosocial adjustment, in childhood and adolescence, presents certain inconsistencies with regard to the influence of these parenting styles on children’s psychosocial well-being and behavioral adjustment [32,33]. Especially in the Anglo-Saxon culture, many authors have observed that the authoritative parenting style is related to children’s better psychosocial competence, academic competence, and less internalized distress and problem behaviors [31,34]. Moreover, authoritative parents were successful in protecting their adolescents from problem drug use [34] and delinquency [35].

However, when the results refer to the Spanish population, and different European and Latin-American countries, there is no such unanimity, and some studies, in the line of Anglo-Saxon research, point to the authoritative parenting style as the most beneficial. In these research studies, the authoritative style has been linked with better psychological adjustment and resilience [36,37,38,39]. Further, the authoritative style is less related to bullying and physical and verbal aggressive behaviors [36,37,40], whereas others recent studies suggest that the indulgent parenting style is less related to hostility [41], school misconduct, delinquency [42], drug use [42,43,44,45], alcohol and tobacco use [44,45,46,47], and bullying and cyberbullying [44], and obtains better results in psychological adjustment [29], socialization [48], and self-esteem [44].

### 1.3. The Present Study

Although numerous investigations relate parenting styles and violence, very few studies analyze, in detail, the functions of violence, age, and sex and the role of the parenting styles, like that of Rathert et al. [49]. These authors considered the variables indicated in the present study, but with children interned in psychiatric institutions.

The goal of this research is to analyze the relationship between the functions of violence and parenting styles, according to the typology of Maccoby et al. [4] as a function of sex and age in a non-clinical sample of Spanish adolescents. The analyses of these differential variables must be taken into account to develop more specific preventive strategies and interventions.

The fact of using parenting styles rather than parental dimensions is also noteworthy. According to Domenech-Rodriguez et al. [50], the usefulness of parenting styles in scientific analysis and professional practice is their consistency across time and situations, and the precise description of the parental behaviors [4,51,52]. In contrast, the dimensions are organized as an amalgam of parental characteristics that do not accurately reflect the situation studied [51].

The working hypothesis is that adolescents educated in families with authoritative and indulgent parenting styles will obtain significantly lower scores in all functions of violence analyzed, in comparison with adolescents educated in families with authoritarian and neglectful parenting styles. In addition, adolescents from the indulgent group will be the least violent of all the evaluated groups.

## 2. Materials and Methods

### 2.1. Participants

The participants in this study were 2399 adolescents (50.2% boys) that were enrolled in 9 public and concerted schools, which taught compulsory secondary education and high school, in Andalucía (Spain). The ages of the participants ranged from 12 to 18 years (*M* = 14.69, *SD* = 1.82). For sample selection, we used randomized group sampling in the geographical area of Andalusia. The primary sampling units were the geographical urban and rural areas. The subunits were the concerted and public schools in each area, which were selected randomly and proportionally. A series of prior analyses of mean differences in the target variables was performed as a function of the location of the school, and whether it was public or concerted, without observing any statistically significant differences.

### 2.2. Procedure

This study is part of a larger national research on violent behavior in adolescence (reference PSI2015-65683-P), which was authorized by the ethics committee of the participant universities and the corresponding educational institutions of the Spanish government in 2016. The study, also, fulfilled ethical values required in research with human beings, respecting the fundamental principles included in the Declaration of Helsinki and its subsequent updates.

The selected schools were initially contacted to explain the goals, the scope of the investigation, and to request their participation. We then sent a letter to the parents of the students, explaining the investigation and requesting their written consent for their children’s participation in the study. After obtaining the corresponding permits, the administration of the instruments was held in one session lasting approximately 45 minutes. Instrument administration was performed under the supervision of previously trained researchers, in the habitual classrooms of each of the participating groups and during a regular class period. The adolescents were informed that their participation in the study was voluntary and anonymous, and that they could withdraw at any time during the process.

### 2.3. Materials

Parenting styles were captured with the involvement–acceptance and control–imposition dimensions of the Parental Socialization Scale (ESPA29) [53]. This instrument was based on the two-dimensional theoretical model of parental socialization [4,46]. It consisted of 212 items (106 parallel items for each parental figure; mother and father). The adolescents rated the actions of their parents in 29 situations that were representative of everyday family life in western culture; 16 refer to the behaviors of the children that are in accordance with the family rules (e.g., “If I respect the schedules established in my home”) and 13 refer to when their behavior goes against these rules (e.g., “If I’m dirty and untidy”). For each of these situations, the adolescents rated, on a 4-point scale ranging from 1 (never) to 4 (always), how their parents act in terms of affection (“Shows me love”) and indifference (“Is indifferent”) in the face of adapted behavior; and in terms of dialogue (“Talks to me”), displeasure (“Doesn’t care”), verbal coercion (“Scolds me”), physical coercion (“Hits me”), and deprivation (“Deprives me of something”) in the face of behavior that disobeys the rule. From these assessments, a global measure in the dimensions of the socialization model—involvement–acceptance and control–imposition—was obtained, through which the parenting style was classified as authoritative, indulgent, authoritarian, and neglectful. The family scores in both orthogonal dimensions (involvement–acceptance and control–imposition) were obtained by averaging the subscales of fathers and mothers. The score in the dimension involvement–acceptance was obtained by averaging the subscales of affection, dialogue, indifference, and displeasure (in the last two, the score is reversed because they are inversely related to the dimension). The score in the control–imposition dimension was obtained by averaging the subscales of verbal coercion, physical coercion, and deprivation. Parenting style groups were formed by using median split procedures. The authoritative parenting style group was those that scored above the median in both dimensions, the indulgent group scored above in the involvement–acceptance dimension and below in control–imposition, the authoritative group scored below in involvement–acceptance and above in control–imposition, and finally, the neglectful parenting style group scored below in both dimensions. The Cronbach alpha reliability coefficients for the scale were: Involvement–acceptance 0.90; and control–imposition 0.96; and for the seven subscales, they were: Affection 0.96; indifference 0.96; dialogue 0.96; displeasure 0.91; verbal coercion 0.95; physical coercion 0.95; and deprivation 0.96.

Reactive and proactive adolescent violence were captured with the Violent Behavior Scale [9]. Based on the multidimensional measurement of self-reported violent behavior developed by Little et al. [9], the 30-item scale contains three Likert-type subscales that assess pure violence, reactive violence, and proactive violence on a 4-point Likert scale, ranging from range 1 (never) to 4 (always). The first scale—pure violence—refers to violence, both overt and relational, independently of their function (e.g., “I am a person who fights with others”, “I am a person who treats others with indifference or stops talking to them”). The second scale—reactive violence—evaluates overt and relational violent behavior as a response to the perception of a previous assault, that is, the reactive function of violence (e.g., “When someone hurts me or injures me, I hit them”, “When someone annoys me, I gossip or spread rumors about that person”). The third scale—proactive violence—measures overt and relational violent behaviors that are used as a means to achieve an end, that is, the instrumental or proactive function of violence (e.g., “I hit, kick, or punch to get what I want”, “To get what I want, I don’t let some people be part of my group of friends”). The Cronbach alpha reliability coefficients obtained for these three scales in our data were 0.64, 0.68, and 0.77, respectively.

### 2.4. Data Analyses

The average of missing data was 2.1%, and never above 5% for an individual measure. The low level of missingness meant that it was not likely to bias the results, thus the estimations were accurate to the expected values on the population [54]. Missing values by scales or subscales were processed using the regression imputation method. In this method, rows in the data matrix were presumed to constitute a random sample of a normal multivariate population. Univariate outliers were detected via the exploration of standardized scores. Following the criteria provided by Hair et al. [55], atypical values were those whose standardized scores had an absolute value above 4. For multivariate detection, Mahalanobis distance was computed. A multivariate outlier was identified if the associated probability at a Mahalanobis distance is 0.001 or less [56].

We calculated the cross-distribution of parenting styles with sex and age groups. Subsequently, the statistical technique of multivariate analysis of variance (MANOVA) with the statistical package SPSS (version 17) was used. MANOVA is a technique that reveals group differences through the comparison of the means obtained by each group in the dependent variables, and it allows for the comparing of the statistical significance of these differences by considering the equality of the compared means as the null hypothesis.

Prior to examining multivariate effects, multivariate normality, equality of variances, and homogeneity of variance–covariance matrices of MANOVA were checked. We applied a multivariate factorial design (MANOVA, 4 × 2 × 2) with the set of criterion variables (pure violence, reactive violence, and proactive violence), considering the parenting style (authoritative, indulgent, authoritarian, and neglectful), sex (male and female), and age group (12–14 years and 15–18 years) as independent variables, to analyze the possible effects of interaction. Subsequently, we applied univariate F-tests to study differences in the dependent variables and we applied the Bonferroni post-hoc testing.

## 3. Results

### 3.1. Descriptive Analyses

Table 1 shows that the cross-distribution of parenting styles with sex, χ^2^(3) = 9.87, *p* < 0.05, was not statistically homogenous, which implies that the two variables were related to each other. The significant typified residuals (values higher than ± 1.96) showed that there were fewer boys educated with the authoritarian parenting style than expected (r z = −2.6). While, the number of girls educated with the authoritarian style was greater (r z = 2.6). The residuals also indicated a positive relationship between being a boy and being educated with the authoritative parenting style (r z = 2.5), this effect was negative for girls (r z = −2.5). With regard to distribution between age group and parenting styles, the results showed no significant differences between the groups, χ^2^(3) = 4.38, *p* > 0.05, indicating that they are statistically homogeneous.

### 3.2. Prior Multivariate Analyses

The MANOVA carried out with the three violence variables yielded statistically significant differences in the main effects of parenting style, Λ = 0.951, *F*(9, 5794.88) = 13.53, *p* <0.001, ηp^2^ = 0.017; sex, Λ = 0.948, *F*(3, 2381) = 43.40, *p* <0.001, ηp^2^ = 0.052; and age, Λ = 0.99, *F*(3, 2381) = 7.86, *p* = 0.01, ηp^2^ = 0.01. We also obtained a statistically significant interaction between parenting style, sex, and age (Λ = 0.993, *F*(9, 5794.88) = 1.96, *p* = 0.04, ηp^2^ = 0.002). No statistically significant interactions were obtained between parenting styles and sex (Λ = 0.997, *F*(9, 5794.88) = 0.876, *p* = 0.546, ηp^2^ = 0.001), parenting styles and age (Λ = 0.998, *F*(9, 5794.88) = 0.90, *p* = 0.04, ηp^2^ = 0.001), or sex and age (Λ = 0.999, *F*(3, 2381) = 0.708, *p* = 0.547, ηp^2^ = 0.001).

### 3.3. Main Effects of the Demographic Variables

With respect to the variable sex, the ANOVA yielded statistically significant differences in pure violence, reactive violence, and proactive violence. As shown in Table 2, boys obtained higher scores in pure violence, reactive violence, and proactive violence than girls.

The ANOVA, in which age was considered an independent variable, yielded statistically significant differences in pure and reactive violence but not in proactive violence. Adolescents aged 15 to 18 years obtained higher scores in pure violence and reactive violence than the group of adolescents aged between 12 and 14.

### 3.4. Parenting Style and Violence

The ANOVA yielded statistically significant differences in pure violence, reactive violence, and proactive violence as a function of the parenting styles (see Table 3).

The Bonferroni tests (α = 0.05) indicated that adolescents from indulgent and authoritative families obtained statistically lower scores in pure violence and proactive violence than adolescents who described their parents as authoritarian and neglectful; no significant differences were found between these latter two groups. Regarding reactive violence, adolescents classified in the indulgent group obtained significantly lower mean scores than all the other groups, among which no differences were found.

### 3.5. Interaction Effect between Parenting Style by Sex and Age

**Figure 1:**
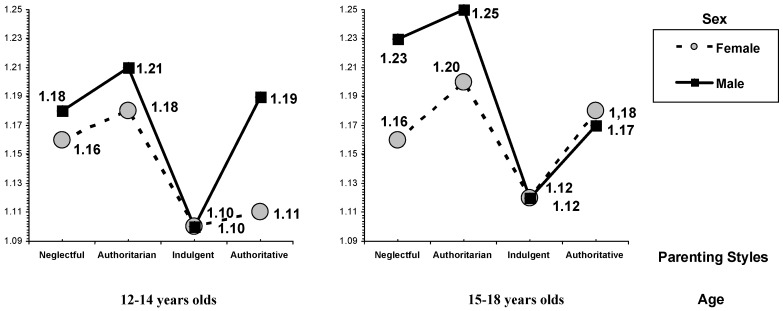
Means of proactive violence by parenting style, sex, and age.

As shown in Figure 1, there was an interaction effect between parenting style, sex, and age in the variable proactive violence, *F* (15, 2383) = 5.19, *p* < 0.001, ηp^2^ = 0.032. However, no significant differences were found in pure violence and reactive violence.

In the Bonferroni test (α = 0.05), we found that the two groups of male adolescents aged 15 to 18 years, from authoritarian and neglectful families, obtained higher scores in proactive violence than the boys and girls from indulgent families, in the two age intervals analyzed (12 to 14 and 15 to 18 years), and also than girls aged 12 to 14 years from authoritative families. We also found higher levels in proactive violence in boys aged 12 to 14 years from authoritarian families compared with girls aged 15 to 18 from indulgent families.

Lastly, the neglectful parental socialization group made up of girls aged 15 to 18 years obtained lower scores in proactive violence in comparison with boys aged 15 to 18 years who rated their socialization as authoritarian and neglectful.

## 4. Discussion

The aim of this research was to explore the relationship between different functions of violence and the parenting styles proposed by Maccoby et al. [4], sex, and age in a sample of Spanish adolescents. In the results obtained in the MANOVA, statistically significant differences were observed in the main effects of parenting style and sex. Regarding the variable sex, girls obtained lower scores in pure violence, reactive violence, and proactive violence than boys. The results also indicated a main age effect, such that adolescents from 15 to 18 years obtained higher scores in pure violence and reactive violence but not in proactive violence. In addition, one of the most interesting results was the effect of the interaction between parenting style, sex, and age in relation to proactive violence.

More specifically, with regard to parenting styles, it was observed that adolescents from indulgent families obtained lower scores in all three dimensions of violence compared to the other groups analyzed. Authoritatively socialized adolescents scored lower in pure violence and reactive violence than the group of neglectful socialization, whereas in proactive violence, there were no differences between the two groups. The group of adolescents from an authoritarian parenting style obtained the highest scores in all the dimensions of violence compared to the adolescents from indulgent and authoritative parenting styles.

The interpretation of these results indicates that higher levels of violence are associated with negative styles of socialization, such as the authoritarian and the neglectful styles. In this sense, in a review on bullying carried out by Álvarez-García et al. [22], two components were identified as risk factors for adolescents to become bullies: Low control of child behavior and low perception of parental support and affective involvement. These characteristics define the neglectful parenting style, and other authors also indicated that this type of parenting style is more frequent in school bullies [17]. However, in contrast to the previous statement, different researchers have highlighted the authoritarian parenting style as the most frequent among violent adolescents [57,58].

The findings of the present study, in which we note that the authoritarian and neglectful parenting styles are the most closely related to violence, contribute, to some extent, to clarify the differences commented on in the afore-mentioned investigations. In this sense, the authoritarian and neglectful parenting styles have in common the parents’ scarce emotional involvement. When considering this aspect, and examining the data in more depth, it is feasible to hypothesize that the absence of emotional support by parents is a key dimension in adolescents’ engagement in violent behavior, perhaps even more so than parental control of behavior. This may be especially true if such control is coercive and unreasoned, as in the authoritarian parenting style.

This hypothesis becomes more relevant when observing that adolescents educated under an indulgent parenting style, in which behavioral control is not restrictive and socialization is done through guidance and affective involvement, are the least violent in comparison to all the other groups analyzed. This result is convergent with those obtained in other studies that relate the indulgent parenting style with the better psychosocial adjustment of adolescents [29,42,44,48]. A socialization style that lacks love may contribute to the development of poor coping resources and social skills, which promotes peer violence.

In reference to the interaction effect among parenting styles, sex, and age, we found significant differences in the variable proactive violence, but nonsignificant differences in reactive and pure violence. Specifically, it was observed that boys aged between 15 and 18 years from authoritarian and neglectful families obtained the highest scores in proactive violence, compared with boys and girls of both age groups from indulgent families, and also than girls aged 12 to 14 from authoritative families.

In addition, we found higher scores in proactive violence in boys aged 12 to 14 years from authoritative families in comparison with girls aged 15 to 18 years from indulgent families. The analysis of the data also reflected less proactive violence in girls aged 15 to 18 years from neglectful families in relation to boys aged 15 to 18 from authoritarian and neglectful families.

This finding reinforces the result obtained in the previously mentioned main effects of parenting styles with regard to the relevance of emotional involvement, but it adds a nuance, suggesting a greater likelihood for boys aged 15 to 18 years from an authoritarian or neglectful environment to use proactive violence.

In this sense, it can be concluded that the relationship between parenting style and proactive violence is complex, and that the role of the variables sex and age must be taken into account. It is feasible to consider that adolescents educated in authoritarian and neglectful households, due to possible habituation to violence in some cases and to social learning, justify and normalize aggressive behaviors in their peer relationships [59]. In this sense, some authors found that adolescents who suffered psychological aggression by their parents were more likely to commit proactive aggression than reactive aggression [60].

In addition, being a male in such families (authoritarian and neglectful) and having passed the phase of early adolescence (12–14 years), together with these models of scarce emotional involvement, increases the likelihood of engaging in proactive violent behavior. The scientific literature has consistently associated boys with greater engagement in violent behavior [61,62,63]. Many of these violent behaviors are limited to traditional masculine gender stereotypes, characterized by relationships of dominance and submission [64], which can be acquired to a great extent in these authoritarian and neglectful family scenarios.

Age also intervenes with parenting style and sex in relation to proactive violence. In a meta-analysis of bullying performed by Tsaousis [8], it is stressed that, as age increases, sensitivity to behaviors that involve intimidation decreases. The increased exposure to violent dynamics undergone by some adolescents from an early age, both in the family and in the classroom, contributes to their developing dominant, competitive, and aggressive attitudes, and they yearn to be perceived as emotionally strong. These features can also involve proactive violence, which, in turn, has been linked to delinquency problems, leadership skills, social reputation, and competence [12].

From a positive viewpoint, it is important to note that, in the interaction effect analyzed, the indulgent parenting style, both in boys and girls and in all of the age ranges considered, obtained lower scores in proactive violence. Social learning acquired in indulgent family scenarios encourages the development of other positive strategies, mainly based on affection, to resolve problems instead of resorting to proactive violence. That is, high emotional involvement, positive communication, parental support, low imposition, and trust between parents and children would develop multiple resources in these adolescents, such that violence would not be a means to achieve their goals.

However, due to the few studies conducted in this area, on parenting styles and the functions of peer violence, taking into account age and sex, we consider that the topic deserves more exploration. Mainly, longitudinal studies are needed to deepen the analysis of potential mediators in the relationship between parenting styles and school violence, and to establish temporal associations. In this sense, the results presented in this work should be interpreted with caution due to the cross-sectional and correlational nature of the data, so we cannot establish causal relationships among the variables. In addition, the use of self-reports requires certain reservations with regard to the information collected. However, to acquire a better understanding of this period and of adolescents’ experience, we must consider their own subjectivity [65]. In fact, adolescents’ experience is validated and valued in the social and cultural context in which they live [66].

It is also necessary to refer to the effect sizes of this study, which can be regarded as small. However, Pinquart [2], in an extensive meta-analysis on parenting styles and externalizing problems in children and adolescents, stated that, although the effects observed in numerous studies are very small, they have practical relevance.

## 5. Conclusions

Finally, we note the importance of promoting parental practices that are not authoritarian and are based on affection and warmth, which contribute to the well-adjusted psychosocial behavior of children and their lower engagement in violent behavior. It is significant to note that this communitarian study is fully in line with other Spanish research studies that have indicated similar facilitators and barriers to the implementation of an evidence-based parenting intervention to efficiently contribute to positive parenting practices [19,20,67,68]. In addition, the role of sex and age must be taken into account because of the complexity of the relationship between proactive violence and parenting styles. Therefore, future research and prevention and intervention models must consider these variables and examine, in greater depth, the relationship between them. In terms of selecting appropriate intervention models, it is important for practitioners to consider a social–ecological perspective for the development of prevention and intervention strategies for reducing violence among adolescents. This aspect suggests the need to consider parental education in prevention programs. Effective programs include the families’, and their children’s, implication and active participation, which in turn will promote positive school coexistence and reduce violent behavior. It is especially important that families will encourage prosocial behaviors in their children. This change implies parenting skills, warmth, and understanding to enhance and support parent–child relationships. From this point of view, the indulgent parenting style is key to learn to resolve conflicts in a socially-desirable way in adolescence.

## Figures and Tables

**Table 1:** Sociodemographic variables.

Variables	Total Sample *N* (%)	Parenting Styles				
		Neglectful (*n* = 736) *N* (%)	Authoritarian (*n* = 513) *N* (%)	Indulgent (*n* = 457) *N* (%)	Authoritative (*n* = 693) *N* (%)	χ^2^
Sex						χ^2^(3) = 9.87 *
Boys	1204 (50.2%)	372 (30.9%)	231 (19.2%)	226 (18.8%)	375 (31.1%)	
Girls	1195 (49.8%)	364 (30.5%)	282 (23.6%)	231 (19.3%)	318 (26.6%)	
Age Group						χ^2^(3) = 4.38
[12,13,14]	1144 (47.7%)	344 (30.1%)	228 (19.9%)	228 (19.9%)	344 (30.1%)	
[15,16,17,18]	1255 (52.3%)	392 (31.2%)	285 (22.7%)	229 (18.2%)	349 (27.8%)	

Note: * *p* < 0.05.

**Table 2:** Means, standard deviations, and differences on violence by sex and age.

Type of Violence	Sex				Age			
	Boys *M* (*SD*)	Girls *M* (*SD*)	*F* (1, 2383)	η^2^p	12–14 *M* (*SD*)	15–18 *M* (*SD*)	*F* (1, 2383)	η^2^p
PV	1.38 (0.29)	1.32 (0.25)	29.89 ***	0.012	1.32 (0.27)	1.37 (0.27)	17.08 ***	0.007
RV	1.77 (0.43)	1.58 (0.36)	129.52 ***	0.052	1.65 (0.42)	1.70 (0.40)	11.15 ***	0.005
PRV	1.18 (0.26)	1.14 (0.22)	19.01 ***	0.008	1.15 (0.24)	1.16 (0.24)	0.90	0.000

PV: Pure violence; RV: Reactive violence; PRV: Proactive violence. * *p* < 0.05. ** *p* < 0.01. *** *p* < 0.001.

**Table 3:** Means, standard deviations, and post-hoc comparisons between parenting styles, pure violence, reactive violence, and proactive violence.

Type of Violence		Parenting Styles					
	Neglectful *M* (*SD*)	Authoritarian *M* (*SD*)	Indulgent *M* (*SD*)	Authoritative *M* (*SD*)	*F* (3, 2383)	ηp^2^	Post Hoc
PV	1.38 (0.28) ^a^	1.40 (0.28) ^b^	1.27 (0.23) ^c^	1.32 (0.27) ^d^	28.50 ***	0.035	c < a, b, d d < a, b
RV	1.70 (0.39) ^a^	1.74 (0.39) ^b^	1.54 (0.38) ^c^	1.70 (0.44) ^d^	25.96 ***	0.032	c < a, b, d
PRV	1.17 (0.25) ^a^	1.19 (0.26) ^b^	1.10 (0.20) ^c^	1.16 (0.24) ^d^	13.58 ***	0.017	c < a, b, d d< b

PV: Pure violence; RV: Reactive violence; PRV: Proactive violence. α = 0.05. *** *p* < 0.001.
